# Downregulation of enhancer RNA AC003092.1 is associated with poor prognosis in kidney renal clear cell carcinoma

**DOI:** 10.1038/s41598-024-64431-8

**Published:** 2024-06-12

**Authors:** JunJie Li, JingZheng Gan, Chen Chen, Yuan Yuan, Xi Xiong, Lili Li, Pengcheng Luo, Wei Zhang

**Affiliations:** 1 Department of Urology, School of Medicine, Wuhan Third Hospital, Wuhan University of Science and Technology, Wuhan, 430060 China; 2 Department of Urology, Wuhan Third Hospital, Tongren Hospital of Wuhan University, Wuhan, 430060 China; 3 Central Laboratory, Renmin Hospital of Wuhan University, Wuhan, 430060 China; 4 Department of Urology, Wuhan Third Hospital, Wuhan, 430060 China

**Keywords:** Cancer, Oncology, Bioinformatics

## Abstract

Kidney renal clear cell carcinoma (KIRC) is the most common histological type of renal cancer, enhancer RNA plays a significant role in tumor growth, however, it has been less studied in renal cancer. The aim of this study was to investigate the role of eRNA AC003092.1 in KIRC. Clinical and RNA expression data were downloaded from a TCGA database, and performed bioinformatics analysis, including expression level analysis, survival analysis, clinical correlation analysis, immune correlation analysis. We further confirmed the expression level of AC003092.1 between normal and tumor cell, predicted the biological role of AC003092.1 in KIRC, and performed cell proliferation and wound healing assays, followed by GSEA enrichment analysis and western blot to detect the proteins of the enriched pathway. Bioinformatics results showed that AC003092.1 expression was elevated in tumor tissues, and knockdown of AC003092.1 expression inhibited cell proliferation and migration. GSEA and western blot results showed that knockdown AC003092.1 expression alleviated the extracellular matrix (ECM) process in KIRC cell lines. Our study provides evidence that AC003092.1 play an important role in KIRC, and AC003092.1 may promote tumor cell progression by affecting the ECM process during tumor development.

## Introduction

Kidney renal clear cell carcinoma (KIRC) is a malignant tumor derived from renal tubular epithelial cells, which has the highest morbidity and mortality rate among all renal cancers^1,2^. Until now, surgical resection is still the most effective therapeutic intervention for treatment of KIRC^3^. Therefore, there is an urgent need to fully elucidate the molecular mechanism of KIRC and develop effective early diagnosis and treatment strategies.

In the last decade, the rapid advancement and widespread utilization of high-throughput sequencing technologies have revolutionized our understanding of non-coding RNAs. Compelling evidence has emerged, linking the dysregulation of non-coding RNAs to the pathogenesis of several prevalent diseases^4,5^. Within the realm of non-coding RNAs, enhancer RNAs (eRNAs) have garnered significant interest owing to their potential involvement in gene transcription and enhancer functionality, as well as their frequent co-localization with non-coding risk loci associated with diseases^6–8^.

Enhancers are crucial cis-regulatory elements that promote the expression of eukaryotic genes^9,10^. eRNAs are long RNAs, ranging from 500 to 5000 bp, that are produced by enhancer transcription^11^. They can independently activate enhancer activity, or bind to other protein factors that promote enhancer-promoter loop formation, thereby activating the expression of downstream genes^12–14^. Dysregulation of eRNAs can affect biological processes, such as cell cycle and cancer cell growth, or alter the expression of target genes, which suggested that eRNAs may serve as a novel target for KIRC treatment^12,14,15^.

**Figure 1:**
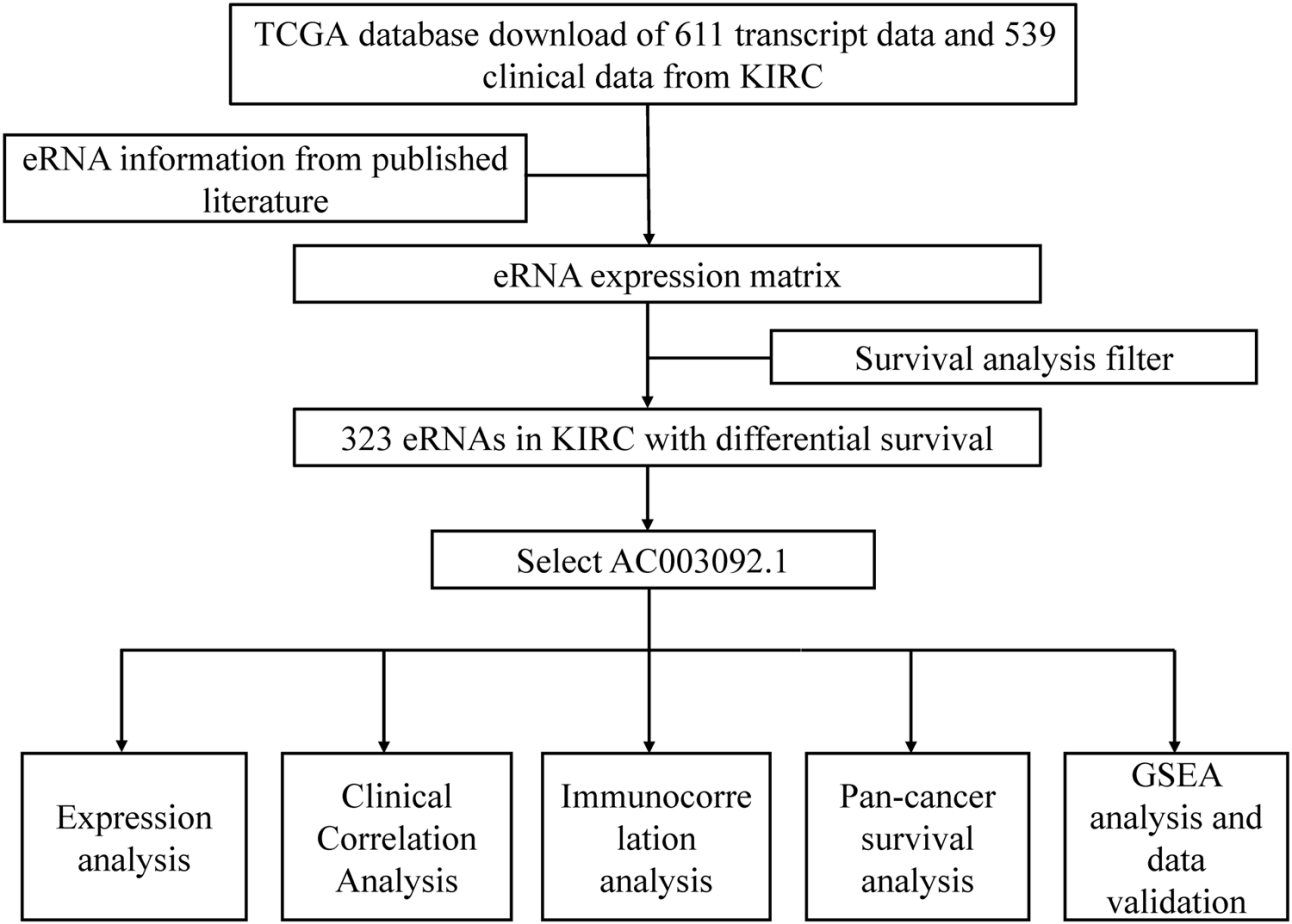
Workflow of this study.

Here, we aimed to identify prognostic eRNAs in KIRC to predict the prognosis of KIRC patients. The results of this study may contribute to a better understanding of the clinical significance of KIRC-associated eRNAs in prognostic stratification and targeted therapy for KIRC. These findings may have the potential to improve clinical practice and patient outcomes. The workflow of this study is shown in Fig. 1.

## Materials and methods

### Identification of eRNA

Clinical data, RNA expression profile data, and survival information for KIRC were downloaded from the TCGA database (https://portal.gdc.cancer.gov/). The eRNA transcript IDs were converted to gene symbols using human GTF files, and the eRNA expression profiles were extracted from the RNA expression profiles of KIRC. We integrated eRNA expression data with survival data from KIRC (Kidney Renal Clear Cell Carcinoma) patients. Survival analysis was performed using the survival package to identify eRNAs that showed significant associations with overall survival in KIRC patients (p-value < 0.05). Supplementary Table 1 presents the detailed information of these eRNAs.

### Immuno-correlation analysis

Based on the RNA-seq expression matrix of KIRC, we conducted analysis using CIBERSORT and ESTIMATE algorithms in R language. The CIBERSORT algorithm (http://cibersortx.stanford.edu/) was utilized to investigate the differential immune cell infiltration status between the high-risk and low-risk groups across 22 immune cell subtypes. We employed the ESTIMATE algorithm to estimate the levels of stromal and immune cells (stromal score and immune score) within the malignant tumor tissue, as well as tumor purity in KIRC samples, as an exploration of a risk scoring model for immune status stratification. Furthermore, we analyzed the expression status of common immune checkpoint markers between the high-risk and low-risk groups using box plots.

### Pan-cancer validation

RNA-seq data from GTEx were processed uniformly in UCSC xena database (https://xenabrowser.net). RNA-seq data in TPM (text per million reads) format were log2 transformed. We downloaded expression profiles and survival data from the UCSC xena database, including Adrenocortical Cancer (ACC); Breast Cancer (BRCA); Cervical Cancer (CESC); Bile Duct Cancer (CHOL); Colon Cancer (COAD); Large B-cell Lymphoma (DLBC); Kidney Clear Cell Carcinoma (KIRC).

### Single gene set enrichment analysis

We performed gene set enrichment analysis (GSEA) using the gene sets provided by MsigDB (https://www.gsea-msigdb.org) to determine the statistical significance and consistent heterogeneity of molecular pathways between the high-risk and low-risk groups. The GSEA software, downloaded from the official website (https://www.broadinstitute.org/gsea/), was implemented using JAVA programming. We selected “h.all.v7.4.symbols.gmt” and “c5.go.v7.4.symbols.gmt” as reference gene sets. Pathways with a false discovery rate (FDR) q-value less than 0.25 and a p-value less than 0.05 were defined as statistically significant.

### Transfection

Two lncRNA AC003092.1-targeting small interfering RNA (siRNA) and a negative control (Control-siRNA) were designed and purchased from Genecreate Corporation (Wuhan, China). All transfections of cells were performed with Lipofectamine 3000 (Invitrogen, USA). The sequence of the lncRNA-AC003092.1 siRNA-1 is: 5ʹ-GUAAUCCAGCGAAUCUGGA-3ʹ, 3ʹ-UCCAGAUUCGCUGGAUUAC-5ʹ. The sequence of the lncRNA-AC003092.1 siRNA-2 is: 5ʹ-CAGCAAUCAACAUAAUCAA-3ʹ, 5ʹ-UUGAUUAUGUUGAUUGCUG-3ʹ. The sequence of the control-siRNA is: 5ʹ-UUCUCCGAACGUGUCACGUTT-3ʹ, 3ʹ-ACGUGACACGUUCGGAGAATT-5ʹ.

### qRT-PCR validation

Total RNA was extracted from cells using the Omega Total RNA Kit II (Conledig, USA) according to the manufacturer’s instructions. RNA was reverse transcribed into cDNA using Reverse Transcription First Strand cDNA Synthesis Kit (TOYOBO, Japan), and quantitative reverse transcription-polymerase chain reaction (qRT-PCR) was performed using 2× SYBR Green qPCR Master Mix (NoneROX, Servicebio, China). The relative expression levels of genes were normalized with β-actin. The primer sequences are shown below: LncRNA AC003092.1: forward primer: TTAGCAGCAAACCCAGAAC, reverse primer: TGCTGAGGATACATGACGAA; β-actin: forward primer: CCTGGCACCCAGCACAAT, reverse primer: GGGCCGGACTCGTCATAC.

### Wound healing assay

A wound healing assay was employed to evaluate cell migration activity. Transfected kidney cancer cells were placed in 6-well plates. When the cells grew to 90–95% fusion, we scratched the wound with a pipette tip. 24 h later, images of the wound area were captured using a digital microscope at × 10 magnification.

### CCK-8 assay

Cell Counting Kit-8 assay (CCK-8 assay) was used to evaluate the proliferation of the cells. The CCK-8 assay was performed as follows: 6 × 10^3^ cells were seeded into 96-well flat-bottomed plates and incubated at 37 °C for 24 h. The cells were then transfected with the appropriate vectors and incubated for an additional 3 days. Absorbance was measured at 450 nm using an enzyme marker (Bio-Rad, Hercules, CA, USA). All experiments were repeated at least three times to ensure accuracy and reproducibility of the results.

### Cell culture

The KIRC cell lines, namely 769-P, ACHN, 786-O, and Caki-1, along with human kidney proximal tubular epithelial cells (HK-2 cells), were procured from the cell bank at the Chinese Academy of Sciences (Shanghai, China). The cell culture media utilized in this investigation encompassed MEM, DEME/F12, and 1640, which were procured from Gino (Wuhan, China). The serum utilized was obtained from GIBCO (Thermo, New Zealand).

### Immunofluorescence experiments

Cell immunostaining was conducted by stimulating the capped cultured cells with the indicated reagents, followed by three times washes with ice-cold PBS, fixation in 4% paraformaldehyde, and blocking with PBS containing 10% normal donkey serum. The samples were subsequently incubated with primary antibodies against Col-1 (GB114197-100, Servicebio, China), FN (GB114491-100, Servicebio, China), and P53 (GB15627-100, Servicebio, China), followed by a goat anti-rabbit conjugate (GB25303, Servicebio, China) as the secondary antibody. DAPI staining was performed, and the sections were observed using a confocal laser microscope (Olympus FV1200, Tokyo, Japan). Six fields of view (× 200 magnification) per group were randomly selected from three separate experiments, and the fluorescence intensity was quantified using software Image J.

### Western blot analysis

Western blot assays were conducted on whole cell following previously described protocols^16^. In brief, lysed samples were loaded onto 10% or 12% SDS gels for electrophoresis and transferred onto PVDF membranes (Merck Microporous), then incubated with the primary antibody overnight at 4 °C. The following day, membranes were washed and incubated with HRP (horseradish peroxidase)-labeled goat anti-rabbit IgG (GB25303, Servicebio, China) for 2 h and then visualized by chemiluminescence. Protein expression was evaluated by the ImageJ software, and then normalized to β-actin (AC026, ABclonal, China).

### Statistical analysis

The results were presented as mean ± standard error of measurement (SEM). Statistical analysis was performed using one-way analysis of variance (ANOVA), survival analysis was performed using t-test. Cox regression model was used for univariate and multivariate survival analysis. The Mann–Whitney test was used to assess the significance of differences between two groups in cell samples. *p* < 0.05 was considered as a statistically significant difference. All statistical analyses were performed using Prism 8.0 (GraphPad Software, Inc., USA). Analyses and visualizations were performed using R v4.1.2 (http://www.r-project.org).

## Results

### Screening for key eRNAs in KIRC

**Figure 2:**
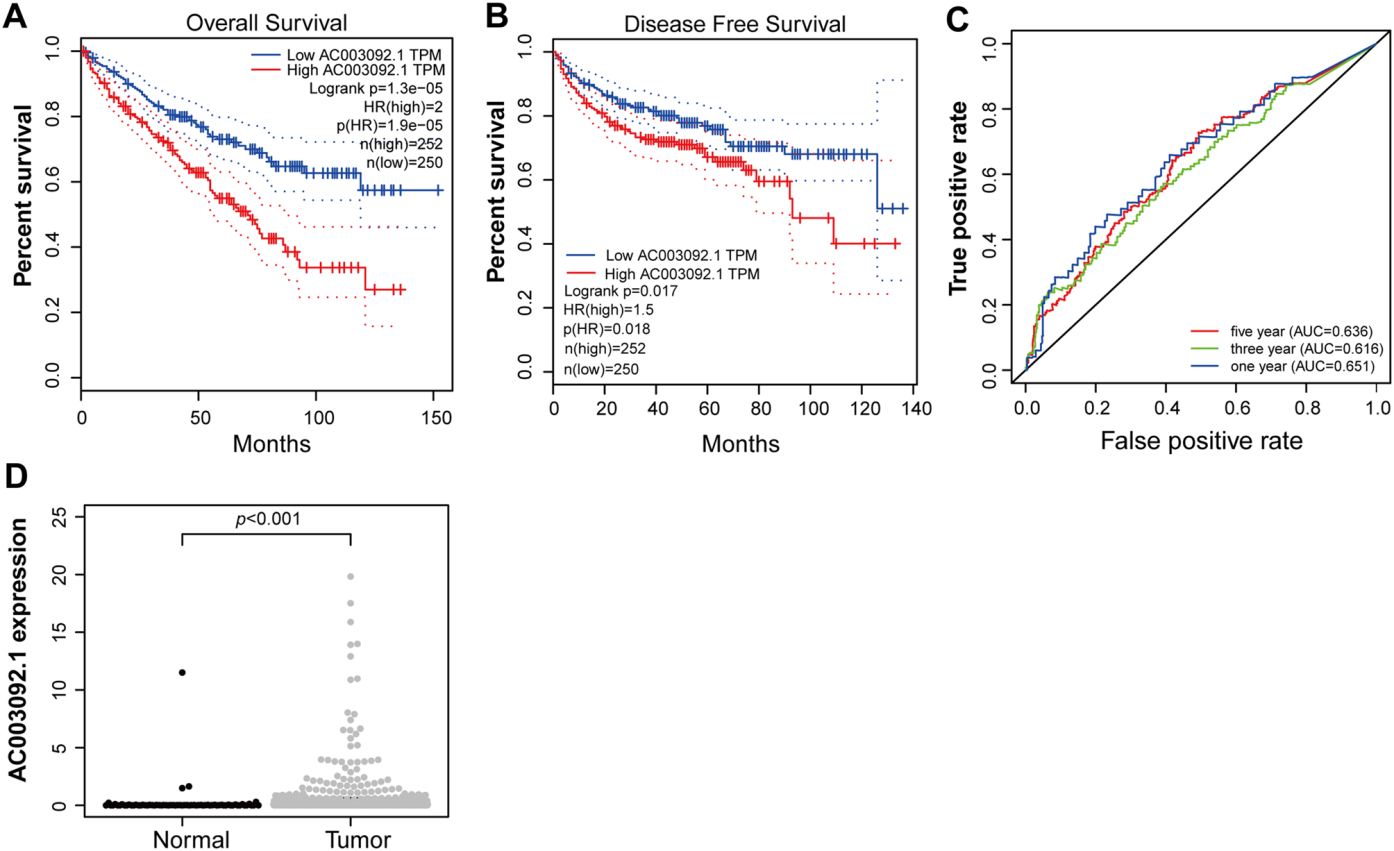
AC003092.1 identified as a KIRC risk gene. (**A**) Kaplan–Meier overall survival curve for AC003092.1. (**B**) Kaplan–Meier curve for disease-free survival for AC003092.1. (**C**) ROC curve of AC003092.1. (**D**) Expression of AC003092.1 in the TCGA database.

We retrieved the expression data of enhancer RNAs (eRNAs) from the TCGA database and integrated it with patient survival data to explore the potential role of eRNAs in kidney renal clear cell carcinoma (KIRC). Through survival analysis, we identified 323 eRNAs that are significantly associated with patient survival in KIRC. The p-values for overall survival of these eRNAs can be found in Supplementary Table 1. eRNA AC003092.1 showed a particularly significant association with overall survival (p < 0.001, Supplementary Table 1). In addition, our validation analysis using the GEPIA database confirmed that patients with high expression of AC003092.1 have significantly lower overall survival rates (Fig. 2A).

Figure 2B showed that elevated AC003092.1 expression in patients with shorter disease-free survival (Fig. 2B). The area under curves (AUCs) in the AC003092.1 expression group at 1, 3 and 5 years were 0.651, 0.616 and 0.636, respectively (Fig. 2C). The expression level of eRNA AC003092.1 is significantly different between normal and tumor in TCGA database (Fig. 2D).

**Figure 3:**
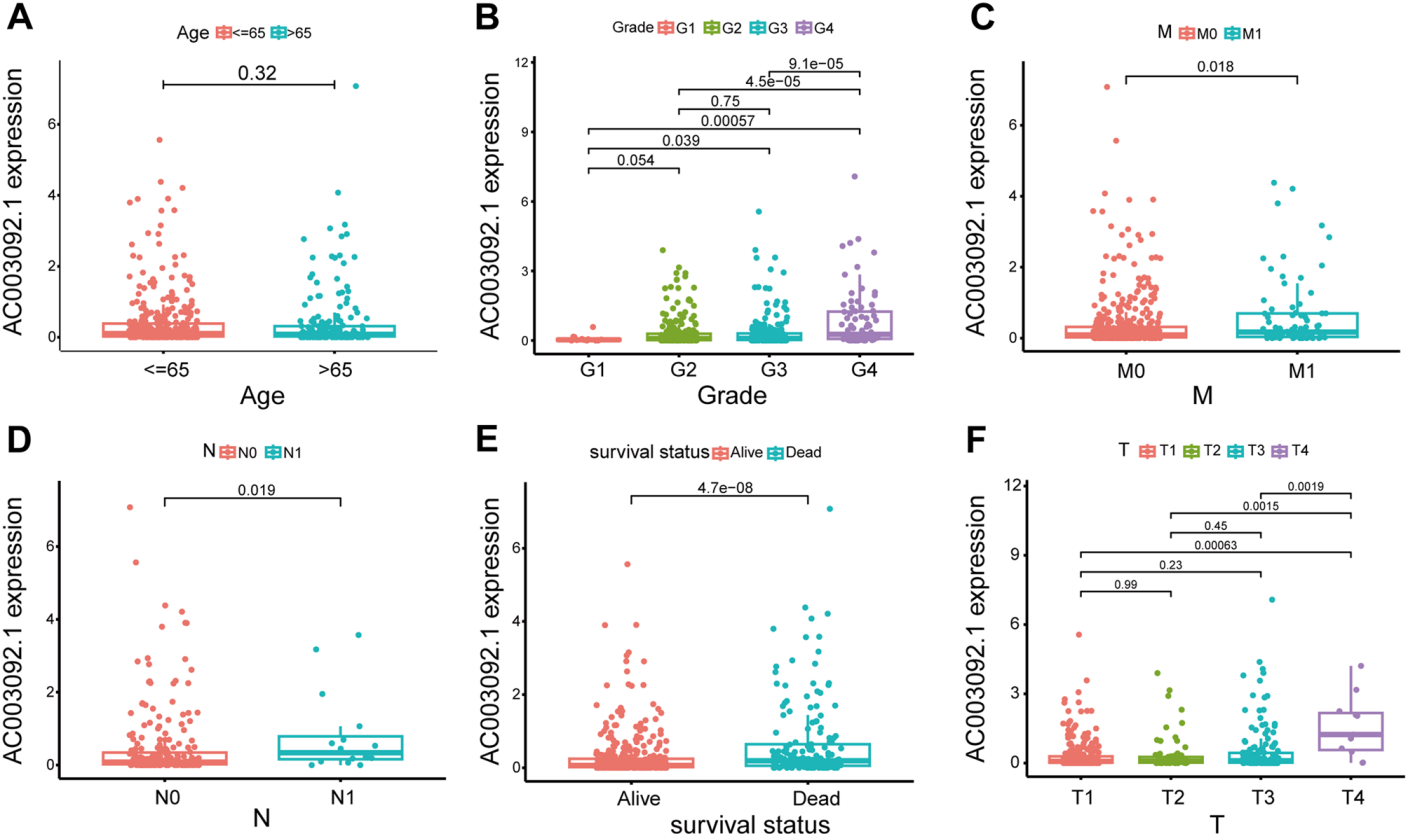
Relationship between AC003092.1 and clinical characteristics. (**A**) Age (*p* = 0.32). (**B**) Grade. (**C**) M stage (*p* = 0.018). (**D**) N stage (*p* = 0.019). (**E**) Survival status (*p* < 0.001). (**F**) T stage.

Additionally, we analyzed the correlation between AC003092.1 expression and clinical characteristics of KIRC patients, and found that AC003092.1 expression levels were significantly associated with age (Fig. 3A), grade (Fig. 3B), M (Fig. 3C), N (Fig. 3D), survival status (Fig. 3E), and T stage (Fig. 3F), which indicated that the expression level of AC003092.1 is associated with clinical parameters.

### Construction and validation of prognostic NOMO graphs

**Figure 4:**
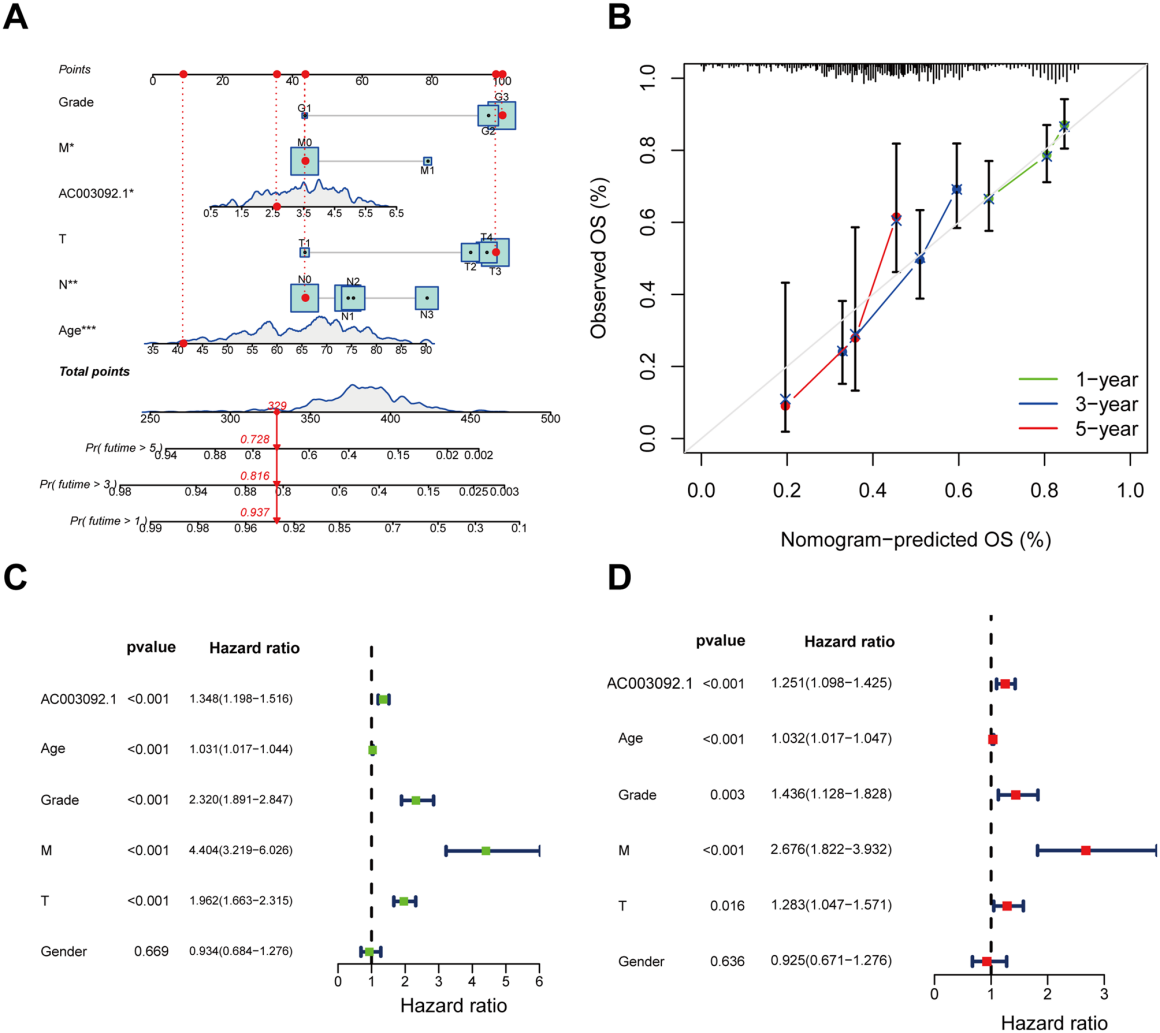
Nomogram construction and validation. (**A**) Column plots of predicted overall survival with age, T-stage, M-stage, clinical stage, sex, and AC003092.1 expression level as parameters. (**B**) Calibration curves for nomograms predicting 1-year, 3-year, and 5-year overall survival. (**C**) Univariate and (**D**) multifactorial Cox regression analysis to determine AC003092.1 as an overall independent clinical feature.

Based on the data from TCGA, we generated Nomogram graphs to predict the overall survival (OS) of patients with KIRC at 1, 3, and 5 years, respectively. The final parameters included AC003092.1 expression level, age, T stage, and M stage (Fig. 4A). The results indicated that the standard curves for 1, 3, and 5 years fitted the ideal model well (Fig. 4B). Furthermore, we conducted univariate and multivariate Cox regression analyses to evaluate the independent prognostic significance of AC003092.1 in KIRC, along with other clinical characteristics such as age, T stage, M stage, and grade. The results of both univariate and multivariate Cox regression analyses showed that AC003092.1 was an independent prognostic factor for OS, with hazard ratios (HRs) of 1.348 (95% confidence interval [CI] 1.198–1.516) and 1.251 (95% CI 1.098–1.452), respectively (Fig. 4C,D).

### Analysis the correlation between eRNA and immune cells

**Figure 5:**
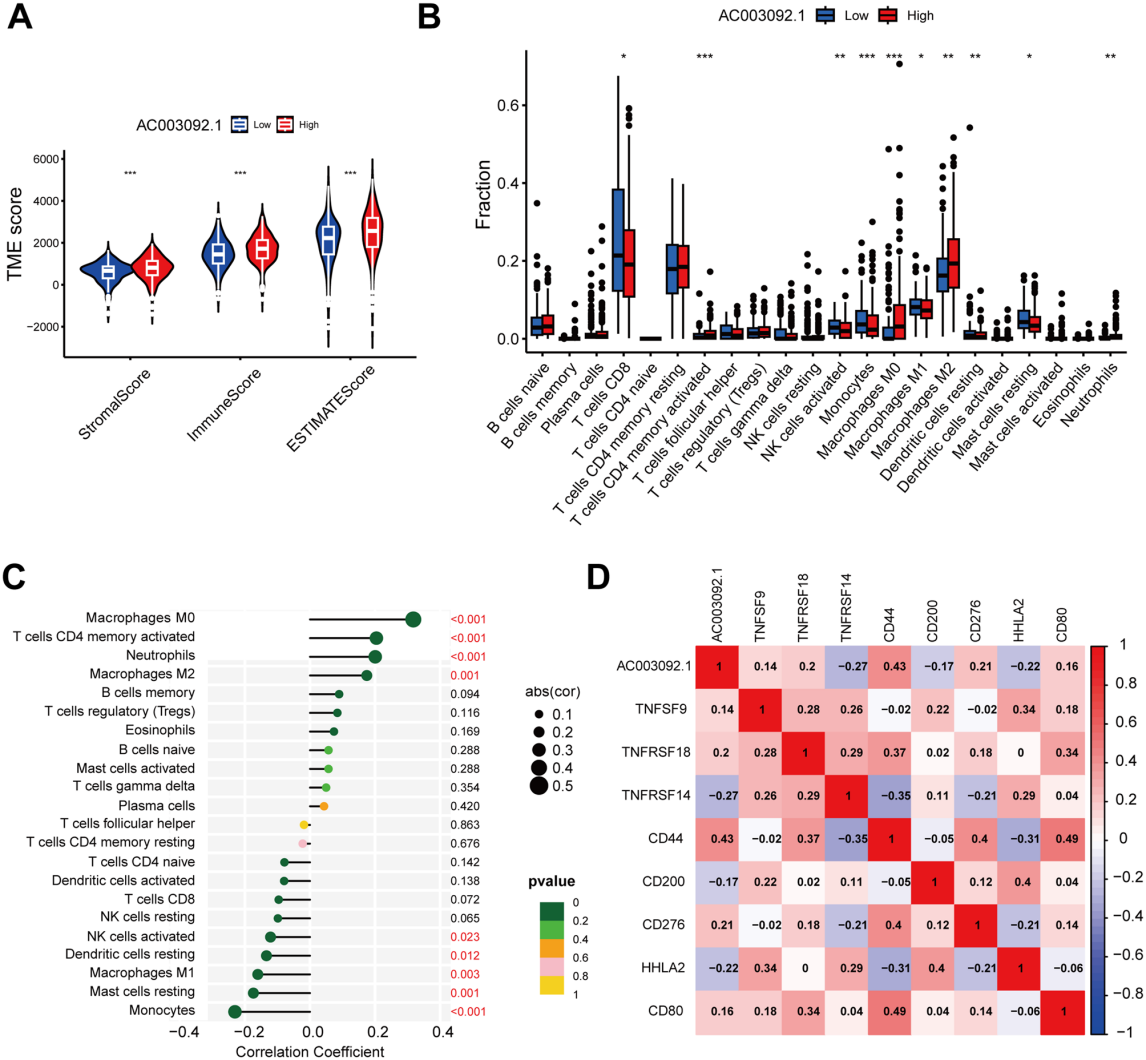
Correlation of AC003092.1 with immune infiltration. (**A**) TME scores at different AC003092.1 expression levels. (**B**) Differences in the abundance of 23 immune cells in the two groups with high and low expression of AC003092.1. (**C**) Correlation between the expression of 23 immune cells and AC003092.1. (**D**) Correlation between AC003092.1 and immune checkpoint-related genes.

To investigate the correlation between the expression level of AC003092.1 and immune cells, we classify the KIRC patients to high and Low groups, based on the median of expression level of AC003092.1. We further utilized the CIBERSORT algorithm to assess the tumor microenvironment (TME) scores of AC003092.1, and the content of immune cells. Our findings revealed a significant correlation between AC003092.1 and TME scores, including Stromal scores. Immune scores and ESTIMATE scores, as shown in Fig. 5A. Furthermore, we analyzed the relationship between the types of 23 immune cells and AC003092.1 expression (Fig. 5B). Our results showed that the levels of M0 macrophages, CD4 memory activated T cells, neutrophils, and M2 macrophages infiltration were positively correlated with the expression of AC003092.1, and the levels of NK cells activated, dendritic cells resting, macrophages M1, mast cells resting, and monocytes infiltration in tumors were negatively correlated with the expression level AC003092.1 (Fig. 5C). Additionally, our study investigated the correlation of eRNA AC003092.1 with 8 of 31 immune checkpoint-related genes. Our results demonstrated that AC003092.1 was positively correlated with TNFSF9, TNFRSF18, CD44, CD276, CD80, and negatively correlated with TNFRSF14, CD200, HHLA2 (Fig. 5D). The above results indicated that eRNA AC003092.1 were associated with cancer immunity.

### Validation of eRNA in pan-cancer

**Figure 6:**
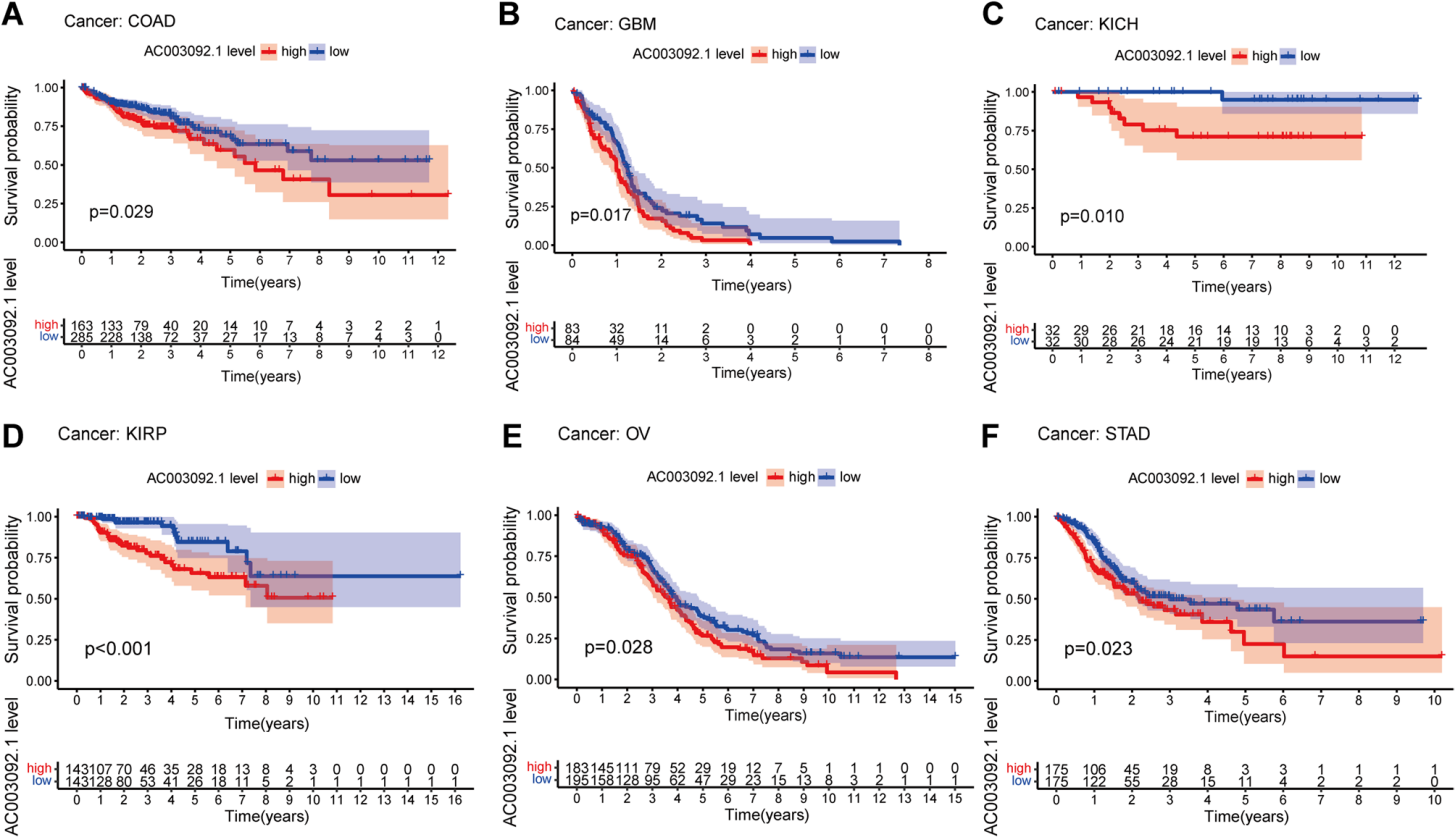
Kaplan–Meier survival curves of AC003092.1 in pan-cancer (p < 0.05). (**A–F**) Survival analysis of AC003092.1 in COAD, GBM, KICH, KIRC, KIRP, OV, and STAD.

Enhancer RNAs (eRNAs) possess transcriptional regulatory functions and play a crucial role in the development of various tumors^17,18^. Therefore, we further analyzed the prognostic significance of the eRNAs AC003092.1 in other types of tumors. Our findings revealed that AC003092.1 was significantly associated with survival outcomes in seven distinct tumor types, including COAD, GBM, KICH, KIRC, KIRP, OV, and STAD. The survival curves of AC003092.1 in these seven tumors are presented in Fig. 6A–F.

### Data validation of eRNA

**Figure 7:**
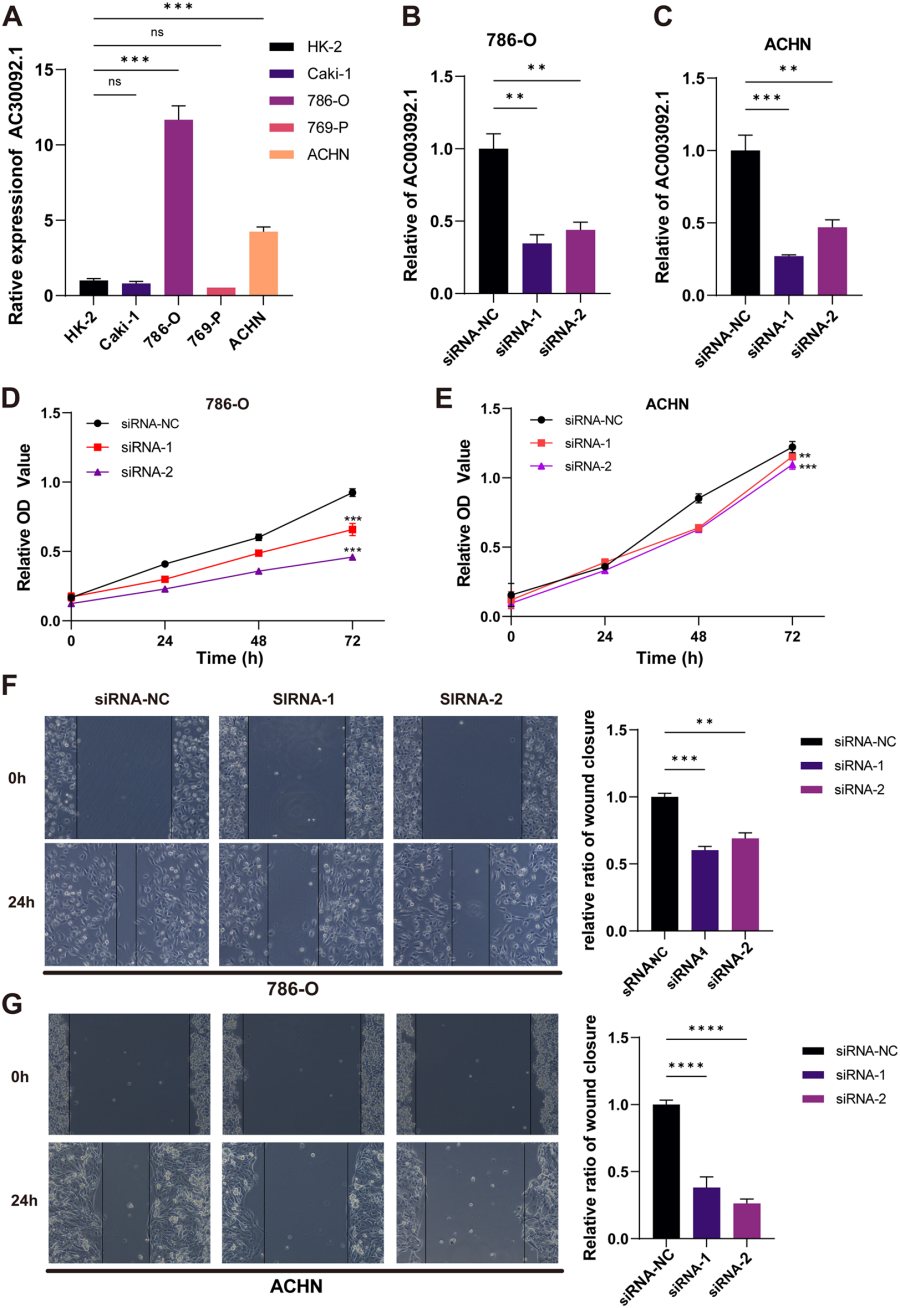
Knockdown of AC003092.1 inhibits cell proliferation and migration. (**A**) q-RTPCR validation of AC003092.1 expression in several cell lines. (**B,C**) q-RTPCR validation of AC003092.1 expression in 786-O and ACHN cell lines. (**D,E**) CC-K8 verifies the proliferation of 786-O and ACHN cell lines. (**F,G**) Wound healing assay designed to assess the effect of AC003092.1 knockdown on cell migration.

Subsequently, the expression level of AC003092.1 was validated in three tumor cell lines, namely 786-O, 786-P, and ACHN, and exhibited elevated AC003092.1 expression level in 786-O and ACHN (Fig. 7A). To investigate the functional role of AC003092.1 in KIRC, siRNA targeting AC003092.1 was transfected into 786-O and ACHN cell lines, respectively. The efficacy of both siRNAs was confirmed using qRT-PCR (Fig. 7B,C). The CCK-8 assay demonstrated that knockdown of AC003092.1 increased the proliferation of KIRC cells in vitro (Fig. 7D,E). Furthermore, the wound healing assay revealed that knockdown of AC003092.1 suppressed the tumor cells invasive activity in 786-O and ACHN cells, respectively (Fig. 7F,G).

### Cell cycle and ECM were regulated

**Figure 8:**
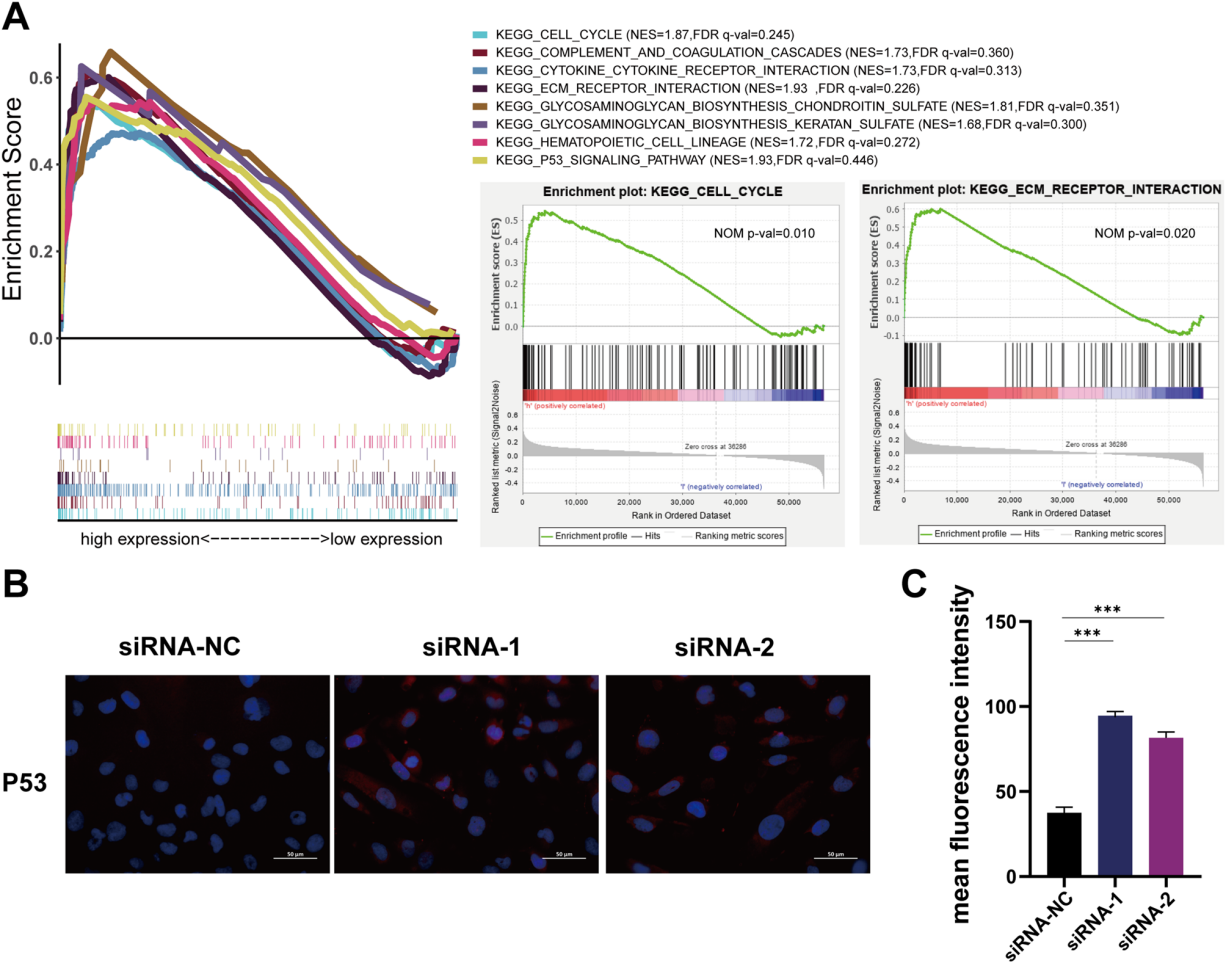
GSEA enrichment analysis. (**A**) GSEA enrichment analysis showed that the high expression group focused on Cell cycle and ECM receptor pathway. (**B,C**) Immunofluorescence showed that the expression level of P53 decreased after knockdown of AC003092.1.

**Figure 9:**
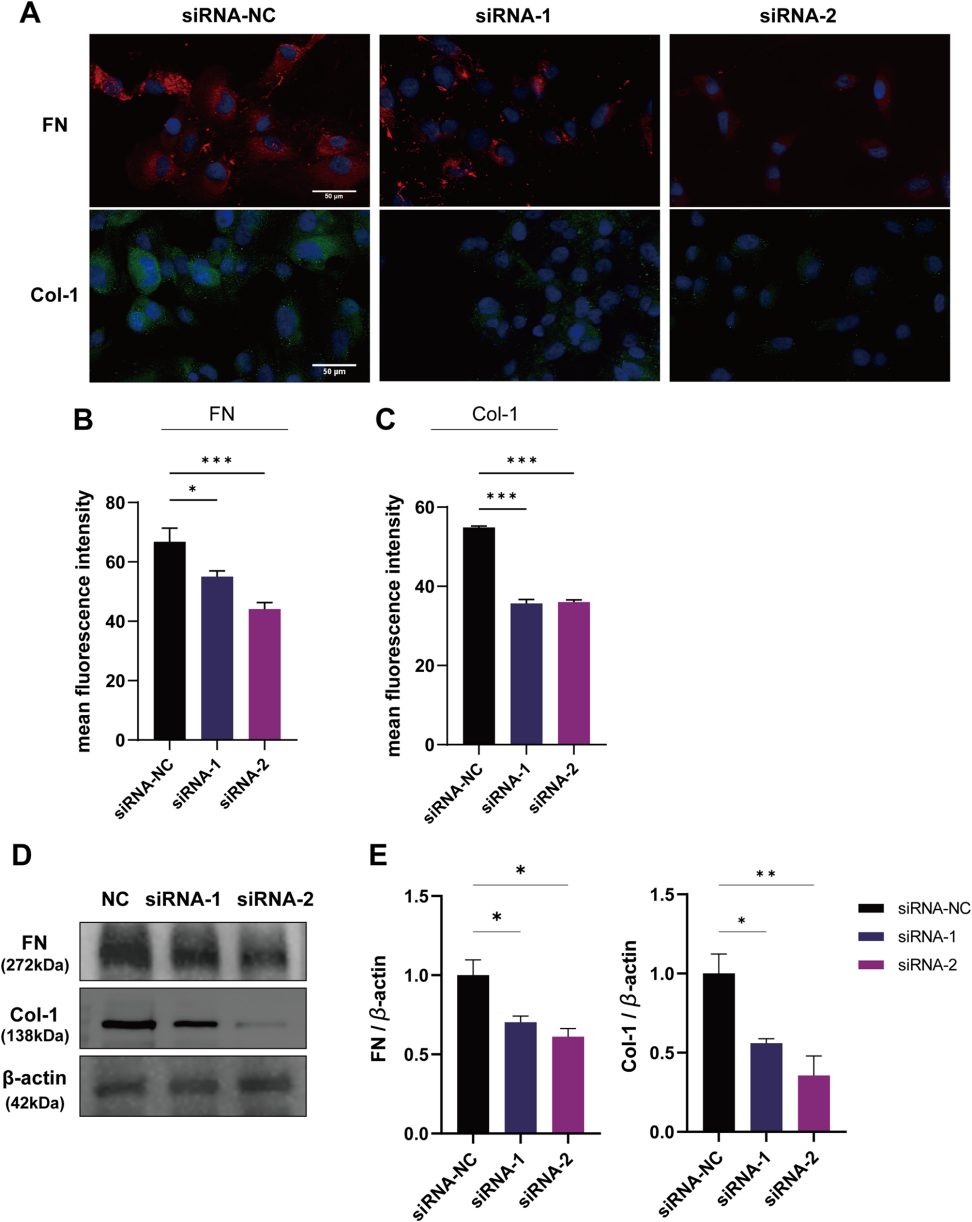
Slowing down of ECM process after knockdown of AC003092.1. (**A–C**) Immunofluorescence shows that FN and Col-1 expression is reduced after knockdown of AC003092.1. (**D,E**) Western blotting experiments showed that FN and Col-1 protein expression levels were reduced after knockdown of AC003092.1.

Knockdown of lncRNA AC003092.1 suppressed the ECM in renal cancer cells. We conducted a single gene enrichment analysis of eRNA AC003092.1, and observed that two gene sets, namely Cell cycle and ECM (*p* < 0.05, FDR qval < 0.25), were enriched in the high-risk group, as depicted in Fig. 8A. Furthermore, a study conducted by Melo et al. revealed that TP53-induced enhancer RNAs (eRNAs) are involved in p53-mediated cell cycle arrest in multiple cancer cell lines^15^.Subsequently, we evaluated the protein expression level of P53 in the 786-O cell line, and found that an increase of the protein expression of P53 after the AC003092.1 knockdown (Fig. 8B,C). In addition, knockdown of AC003092.1 resulted in decreased expression levels of FN and col-1 proteins (Fig. 9A–D), which are two key markers during ECM process. These findings suggested that inhibition of AC3003092.1 expression leads to reduced levels of ECM in tumor cells.

## Discussion

ERNA is a type of non-coding RNA, that is transcribed from enhancer regions. eRNA has been shown to play a significant role in the activation of oncogenes and aberrant signaling pathways, which contribute to tumorigenesis^19,20^. Several studies have demonstrated that oncogene-induced eRNAs can directly promote tumorigenesis. For instance, the androgen-inducible eRNA regulatory gene KLK3e can scaffold androgen receptor (AR)-associated protein complexes to regulate receptor-dependent gene expression in prostate cancer^21^. Conversely, tumor suppressors can also induce eRNA to promote tumor suppressive processes. For example, TP53-induced eRNA has been implicated in p53-dependent cell cycle arrest in various cancer cell lines. These findings suggest that eRNA may serve as a potential therapeutic target for cancer treatment lines^15^.

In previous studies, Guo et al. have reported that AC003092.1 is an immune-related lncRNA and have analyzed its immune correlation with glioblastoma multiforme (GBM)^22^. Our analysis has also revealed the immune correlation of AC003092.1 with KIRC (Fig. 3).

Furthermore, Xu et al. have demonstrated that lncRNA AC003092.1 promotes the drug sensitivity of GBM to temozolomide in a competitive relationship^23^. The function of AC003092.1 in immune cell should be studied further.

In this study, we identified an eRNA AC003092.1, which is associated with KIRC. Subsequent clinical analysis revealed a significant correlation between the expression levels of AC003092.1 and TNM stage and grade stage. Li et al. found that in melanoma, CENPF is closely associated with activated CD4+ T cells and may lead to premature depletion of CD4+ memory T cells and immune suppression^24^. Our studies indicated that AC003092.1 is significantly associated with M0 macrophages, T cells CD4 memory activated, neutrophils in KIRC. Previously, NOMO graph has made great progress in the development of prognostic models for several diseases^25^. The accuracy of the model has been confirmed through the utilization of a standard curve exhibiting high precision. The projected values for the overall survival rate at 1, 3, and 5 years, as determined in this investigation, align consistently with the standard curve, thereby substantiating the reliability of utilizing AC003092.1 for assessing the survival prognosis of KIRC patients.

Some studies have demonstrated that ECM contributes to tumor formation^26,27^. In this study, the validation analysis demonstrated that knockdown of AC003092.1 significantly reduced the proliferation and migration capacity in 786-O cells during ECM formation process. Furthermore, previously Liu et al. highlighted the important role of cell cycle in tumor therapeutic targets^28^, and our data indicated that decreased AC003092.1 level resulted in increased P53 expression, which may imply DNA repair and cell cycle arrest^29,30^.

Our study provides valuable data for studying eRNA in KIRC. Our clinical and immune correlation analysis provides the specific methods to help us to further understand the transcriptional effects of this eRNA in tumors.

## Conclusion

Our study provides a comprehensive analysis of the prognostic significance of eRNA AC003092.1 in patients with kidney renal clear cell carcinoma (KIRC). The results of our research have significant implications for the accurate assessment of patient prognosis, early detection of KIRC, and the development of targeted clinical interventions.

### Supplementary Information

Supplementary Information.

## Data Availability

The data for this study can be downloaded in the UCSC xena (https://xenabrowser.net/), TCGA (https://portal.gdc.cancer.gov/) and GEPIA database (http://gepia.cancer-pku.cn/) and UCSC xena database (https://genome.ucsc.edu).

## Abbreviations

eRNA: Enhancer RNA
KIRC: Kidney renal clear cell carcinoma
GSEA: Gene set enrichment analysis
ACC: Adrenocortical cancer
BRCA: Breast cancer
CESC: Cervical cancer
CHOL: Bile duct cancer
COAD: Colon cancer
DLBC: Large B-cell lymphoma
GBM: Glioblastoma multiforme
