# Supplementary material for: Downregulation of enhancer RNA AC003092.1 is associated with poor prognosis in kidney renal clear cell carcinoma
Source: Sci Rep. 2024 Jun 12;14:13475. doi: 10.1038/s41598-024-64431-8 (PMC11169679; doi:10.1038/s41598-024-64431-8)

**Supplementary Files**

**Supplementary Table 1: eRNA with significant differences survival rate in KIRC.**

| Gene | *P* of overall survival rate |
| --- | --- |
| SNHG17 | 6.82E-12 |
| TMEM92-AS1 | 9.51E-10 |
| TINCR | 2.07E-09 |
| LINC02577 | 1.27E-08 |
| LINC00605 | 2.15E-08 |
| LINC01914 | 2.94E-08 |
| SLC16A1-AS1 | 4.96E-08 |
| AC003092.1 | 1.03E-07 |
| LINC01251 | 1.08E-07 |
| LINC01271 | 1.52E-07 |
| EMX2OS | 3.91E-07 |
| LINC00460 | 5.11E-07 |
| LINC02754 | 5.20E-07 |
| LINC02657 | 5.31E-07 |
| AL391095.2 | 7.78E-07 |
| MIAT | 8.34E-07 |
| LINC00271 | 8.74E-07 |
| AC007879.3 | 1.12E-06 |
| AC128709.2 | 1.29E-06 |
| CYP4F26P | 1.54E-06 |
| AL445524.1 | 1.71E-06 |
| CYP1B1-AS1 | 1.94E-06 |
| LINC01270 | 2.80E-06 |
| LINC00174 | 3.11E-06 |
| CCDC18-AS1 | 4.18E-06 |
| LINC-PINT | 4.70E-06 |
| LILRP2 | 4.72E-06 |
| HOTTIP | 4.88E-06 |
| AC137579.1 | 4.97E-06 |
| LINC01004 | 5.37E-06 |
| C20orf203 | 6.10E-06 |
| AL050327.1 | 6.74E-06 |
| AL023754.1 | 8.62E-06 |
| TCL6 | 9.44E-06 |
| LINC01433 | 9.85E-06 |
| FAM225A | 1.17E-05 |
| ZNF503-AS1 | 1.37E-05 |
| AC105219.1 | 1.44E-05 |
| AL021392.1 | 1.45E-05 |
| AC113346.1 | 1.75E-05 |
| ZMIZ1-AS1 | 2.13E-05 |
| FOXP4-AS1 | 2.14E-05 |
| LINC01671 | 2.54E-05 |
| MIR205HG | 2.68E-05 |
| LINC02810 | 2.68E-05 |
| LINC01293 | 2.85E-05 |
| LINC01060 | 2.90E-05 |
| AC007879.4 | 2.90E-05 |
| AC002451.1 | 2.91E-05 |
| WT1-AS | 3.13E-05 |
| AL356215.1 | 3.13E-05 |
| BX284668.2 | 3.38E-05 |
| AL645608.2 | 3.47E-05 |
| AP003774.2 | 3.68E-05 |
| AC082651.4 | 3.71E-05 |
| LINC01389 | 3.73E-05 |
| LINC01191 | 3.94E-05 |
| NALT1 | 4.09E-05 |
| AC087672.2 | 4.53E-05 |
| OSMR-AS1 | 4.62E-05 |
| LINC02257 | 4.64E-05 |
| HOXB-AS4 | 5.06E-05 |
| LINC02611 | 5.36E-05 |
| THUMPD3-AS1 | 5.36E-05 |
| LINC00323 | 6.03E-05 |
| MOSMO | 6.09E-05 |
| RNF139-AS1 | 6.09E-05 |
| SLC25A24P1 | 7.09E-05 |
| LINC01510 | 7.64E-05 |
| GATM | 7.80E-05 |
| AL118511.1 | 7.88E-05 |
| LINC00607 | 8.07E-05 |
| AC067930.2 | 8.10E-05 |
| JPX | 8.10E-05 |
| STX4 | 8.13E-05 |
| AL359878.2 | 8.50E-05 |
| AC114956.1 | 9.16E-05 |
| LINC01731 | 0.000100742 |
| MIR583HG | 0.000101415 |
| RRP7BP | 0.000102855 |
| LINC02507 | 0.000104188 |
| SSPO | 0.000105077 |
| KCNK15-AS1 | 0.000124005 |
| AC022816.1 | 0.000125496 |
| AL139220.2 | 0.000132244 |
| AC008663.3 | 0.000135234 |
| AC015712.6 | 0.000137522 |
| AC108112.1 | 0.000137913 |
| AP002761.1 | 0.000138016 |
| AC073346.1 | 0.000138764 |
| FAM120AOS | 0.000139687 |
| DLEU2 | 0.00014028 |
| LINC00926 | 0.00015106 |
| FRY | 0.000151522 |
| AL137025.1 | 0.00015763 |
| RPS10P7 | 0.000158853 |
| AL683813.2 | 0.000164549 |
| MIR5689HG | 0.000165882 |
| LINC02569 | 0.00016594 |
| LINC01833 | 0.000168975 |
| LINC01185 | 0.000171964 |
| AP003390.1 | 0.000176147 |
| LINC00160 | 0.000199983 |
| AL359636.2 | 0.000206394 |
| AP003108.1 | 0.000207721 |
| AL358394.2 | 0.000209245 |
| NBPF1 | 0.000218422 |
| AC139491.2 | 0.000220496 |
| LINC00937 | 0.000227714 |
| AP003721.4 | 0.000229763 |
| AL139352.1 | 0.000231214 |
| ARNILA | 0.000232893 |
| BX322234.2 | 0.000240346 |
| LINC01121 | 0.000245118 |
| LINC01522 | 0.000272665 |
| AC133785.1 | 0.000275946 |
| AC007405.2 | 0.000278315 |
| LINC02154 | 0.000296448 |
| HAR1B | 0.000298836 |
| AL928921.1 | 0.000317546 |
| BAALC-AS1 | 0.00032906 |
| AP003174.1 | 0.000331116 |
| LINC01090 | 0.000354543 |
| CDK6-AS1 | 0.000367462 |
| LINC01238 | 0.000368271 |
| LINC02062 | 0.000368549 |
| GARS-DT | 0.000370379 |
| FGGY | 0.000384085 |
| MYOSLID | 0.000418775 |
| AF279873.3 | 0.00042217 |
| AC026785.3 | 0.0004275 |
| FZD4-DT | 0.000429661 |
| GACAT3 | 0.000433289 |
| LINC02832 | 0.000433762 |
| LHFPL3-AS2 | 0.00044453 |
| LINC01133 | 0.000446299 |
| HOTAIR | 0.000460546 |
| WDFY3-AS2 | 0.000473499 |
| ELFN1-AS1 | 0.00048044 |
| LINC00337 | 0.000506079 |
| Z94160.1 | 0.000525062 |
| C2orf92 | 0.00055129 |
| AC010789.1 | 0.000554114 |
| LINC01411 | 0.000559548 |
| EMG1 | 0.000562313 |
| AL021707.2 | 0.000579809 |
| LINC00671 | 0.000582945 |
| AFG3L1P | 0.000591039 |
| SPAAR | 0.000594152 |
| AC012213.1 | 0.000616305 |
| AC010457.1 | 0.000621082 |
| RPL34-AS1 | 0.000639662 |
| LINC01134 | 0.000654385 |
| AL035670.1 | 0.00066467 |
| LINC02614 | 0.00068143 |
| AC073257.1 | 0.000711814 |
| AC008610.1 | 0.000717788 |
| AP002518.1 | 0.00071877 |
| APCDD1L-DT | 0.00072408 |
| LINC00525 | 0.000743774 |
| LINC01176 | 0.000756007 |
| AC093599.1 | 0.000770775 |
| UCA1 | 0.000828521 |
| LINC01486 | 0.00088096 |
| AL390879.1 | 0.000889641 |
| AP000688.3 | 0.000894995 |
| TMEM252-DT | 0.000905262 |
| AL118508.1 | 0.000911629 |
| LINC00261 | 0.00091436 |
| LINC02615 | 0.000922257 |
| AC009022.1 | 0.000960508 |
| AC021074.3 | 0.000985448 |
| AC145285.2 | 0.001033044 |
| LINC01301 | 0.001143996 |
| AC107029.2 | 0.001146744 |
| LINC02455 | 0.001156871 |
| EDRF1-DT | 0.001201765 |
| LINC01891 | 0.001208742 |
| AC008663.1 | 0.001221326 |
| SLC47A1P1 | 0.001320244 |
| LINC00304 | 0.001321935 |
| VPS33B-DT | 0.001381602 |
| HAR1A | 0.001382547 |
| HAGLR | 0.001400552 |
| AC080129.1 | 0.001432069 |
| ZFHX4-AS1 | 0.001459906 |
| LINC02636 | 0.001493626 |
| AP001189.3 | 0.001560969 |
| AP004608.1 | 0.001573242 |
| AC022400.6 | 0.00161583 |
| AC018450.1 | 0.001616892 |
| AC093734.1 | 0.001694035 |
| AC124242.1 | 0.00169981 |
| LINC02275 | 0.001703661 |
| CDKN2B-AS1 | 0.001761882 |
| C1orf61 | 0.001773231 |
| AC092894.1 | 0.001780628 |
| AP001347.1 | 0.001828579 |
| AC006065.4 | 0.001846158 |
| AC012618.3 | 0.001884107 |
| AC068985.2 | 0.001947743 |
| STEAP1B | 0.001956981 |
| DRAIC | 0.001988306 |
| HIVEP2 | 0.002012012 |
| TSGA10IP | 0.002023266 |
| LINC02812 | 0.002033176 |
| MEG9 | 0.002178989 |
| AL391056.1 | 0.002183352 |
| AC006065.3 | 0.002223743 |
| ILDR2 | 0.002232842 |
| LINC02006 | 0.002240194 |
| PTGDS | 0.002274198 |
| AL365259.1 | 0.002282267 |
| TULP4 | 0.00239363 |
| AC084375.1 | 0.002423903 |
| AC078950.1 | 0.00242929 |
| AC103702.2 | 0.002543884 |
| AC226101.1 | 0.002606867 |
| FZD10-AS1 | 0.00269601 |
| MATN1-AS1 | 0.002714757 |
| BPIFB9P | 0.002715701 |
| LINC02087 | 0.002718292 |
| EVX1 | 0.002857089 |
| AC015909.1 | 0.002981306 |
| AC012363.3 | 0.002988344 |
| LRRC37A11P | 0.002989597 |
| AC004917.1 | 0.003042032 |
| LINC01214 | 0.003155239 |
| LINC02331 | 0.003262172 |
| LINC00158 | 0.003292834 |
| RSRP1 | 0.003343173 |
| AC073288.2 | 0.003360292 |
| GNG12-AS1 | 0.003371555 |
| AL157937.1 | 0.003388203 |
| AC114489.2 | 0.003422903 |
| AC093866.1 | 0.003556716 |
| AC093157.1 | 0.003573298 |
| LINC01932 | 0.003579124 |
| AC004941.1 | 0.003631384 |
| LINC02075 | 0.003662042 |
| AC006159.2 | 0.003703781 |
| DCP1A | 0.003794167 |
| LINC02739 | 0.00381321 |
| AL049612.1 | 0.003923516 |
| AC018866.2 | 0.003946613 |
| BLCAP | 0.00396442 |
| AC108067.1 | 0.004031999 |
| RASGEF1B | 0.004139995 |
| LINC02043 | 0.004148595 |
| AC026787.1 | 0.004187032 |
| AC092957.1 | 0.004217834 |
| AC104248.1 | 0.004226084 |
| GCC2-AS1 | 0.004435516 |
| AC022613.1 | 0.004454927 |
| AC004080.1 | 0.004602514 |
| AC004923.4 | 0.004875979 |
| LINC01771 | 0.004876435 |
| AC079760.1 | 0.004902195 |
| AP000251.1 | 0.004942326 |
| LRRC77P | 0.004950916 |
| AL669970.3 | 0.004969779 |
| AC091180.5 | 0.005070828 |
| MSN | 0.005139858 |
| CCDC192 | 0.005323609 |
| AC109492.1 | 0.005450453 |
| AC011352.3 | 0.005530131 |
| LINC02539 | 0.005545067 |
| LINC01795 | 0.00556533 |
| PSG8-AS1 | 0.005621208 |
| AC022424.1 | 0.005632359 |
| ZNF192P1 | 0.005668177 |
| LINC02783 | 0.005724743 |
| SKINT1L | 0.005788366 |
| CYP4A22-AS1 | 0.005822946 |
| AC106795.2 | 0.005871415 |
| AP001476.1 | 0.005935996 |
| AC021028.1 | 0.005969441 |
| AC069281.1 | 0.005987928 |
| AL358876.2 | 0.00608166 |
| AC100849.2 | 0.006313167 |
| EDNRB-AS1 | 0.00643375 |
| AC023794.3 | 0.006550152 |
| LINC02422 | 0.006557017 |
| TPT1-AS1 | 0.006559881 |
| AL353803.2 | 0.006669697 |
| MIR3945HG | 0.006860779 |
| AL355303.1 | 0.006860982 |
| FAM85B | 0.007057548 |
| AC104653.1 | 0.007102169 |
| AP001767.3 | 0.007112004 |
| KANTR | 0.007122783 |
| AL121796.1 | 0.007180583 |
| H1FX-AS1 | 0.007210791 |
| AL034417.3 | 0.007331402 |
| HCG20 | 0.007405004 |
| KCNQ1DN | 0.007447509 |
| AC091435.2 | 0.007468019 |
| AP000880.1 | 0.007470852 |
| LINC01357 | 0.007534343 |
| MEG3 | 0.00758296 |
| AC006206.2 | 0.007652659 |
| LINC00665 | 0.007671703 |
| FOXO3B | 0.007832597 |
| TP53TG1 | 0.008040366 |
| AC124852.1 | 0.008076964 |
| LINC02762 | 0.008100344 |
| AC067930.3 | 0.008182142 |
| LINC00242 | 0.008532355 |
| AC084026.1 | 0.008554339 |
| AP000221.1 | 0.00857714 |
| LINC00601 | 0.008774232 |
| TEX46 | 0.008799504 |
| GAS2L1P2 | 0.008959428 |
| LINC02265 | 0.008976018 |
| LINC01252 | 0.009010139 |
| AC125616.1 | 0.009264216 |
| AC009229.2 | 0.009293701 |
| AC012574.2 | 0.00934537 |
| LINC01850 | 0.009378454 |
| AC005562.1 | 0.009463969 |
| LINC00472 | 0.009498143 |
| AC009226.1 | 0.009744923 |
| MIATNB | 0.009886407 |

Supplementary Figure 1: Original figure of FN’s western blotting experiment:


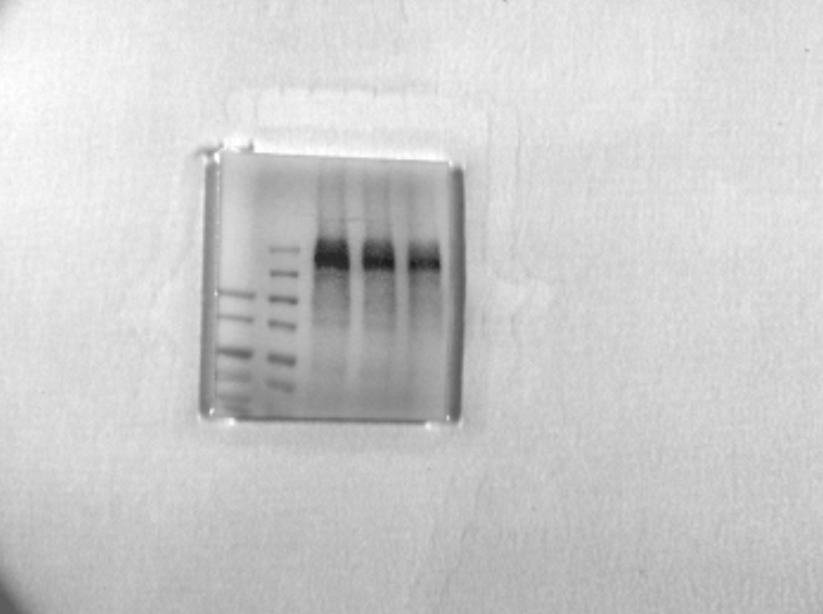


Supplementary Figure 2: Original figure of Col-1’s western blotting experiment:


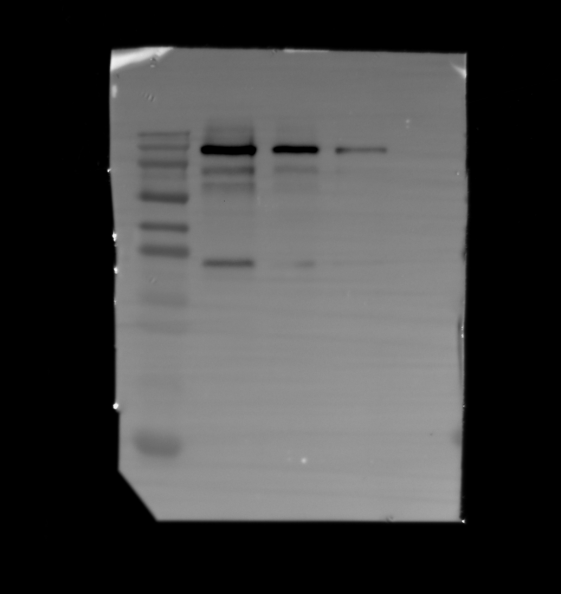


Supplementary Figure 3: Original figure of β-actin’s western blotting experiment:


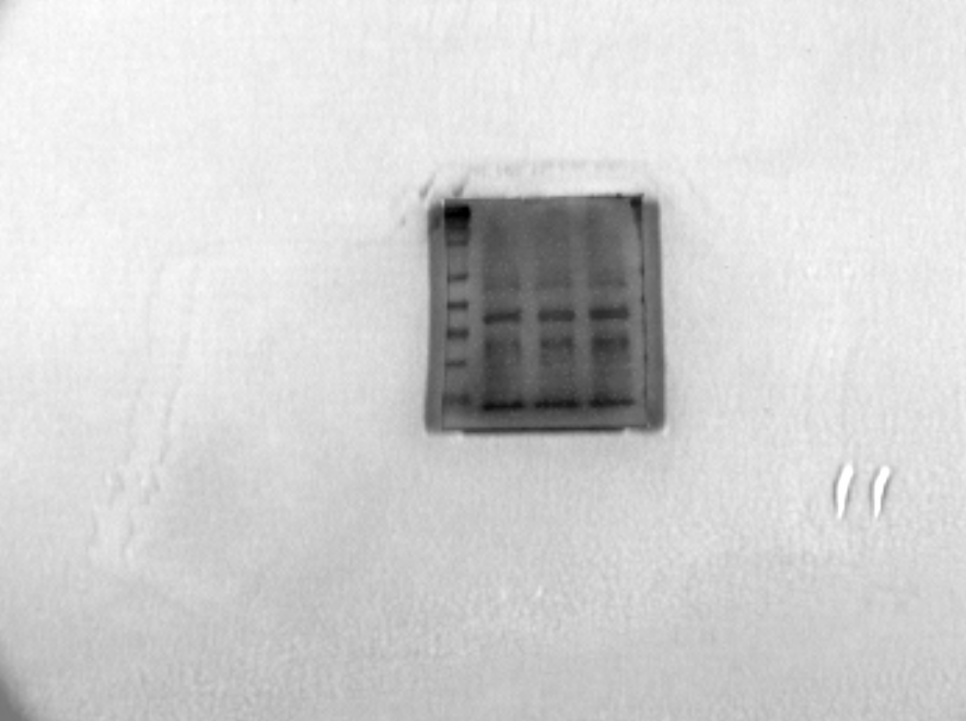

Supplement: Supplementary file 1 — Supplementary Information. [file 41598_2024_64431_MOESM1_ESM.docx]
